# Fertility preservation in ovarian tumours

**DOI:** 10.3332/ecancer.2018.885

**Published:** 2018-12-06

**Authors:** Federica Tomao, Anna Di Pinto, Carolina Maria Sassu, Erlisa Bardhi, Violante Di Donato, Ludovico Muzii, Maria Cristina Petrella, Fedro Alessandro Peccatori, Pierluigi Benedetti Panici

**Affiliations:** 1 Department of Gynaecological and Obstetrical Sciences and Urological Sciences, Sapienza University of Rome, 00161 Rome, Italy; 2 Division of Gynaecologic Oncology, European Institute of Oncology, Milan, Italy

**Keywords:** ovarian cancer survivors, fertility preservation, ovarian cancer, fertility in cancer patients

## Abstract

A considerable number of patients with a cancer diagnosis are of childbearing age and have not satisfied their desire for a family. Despite ovarian cancer (OC) usually occurring in older patients, 3%–14% are diagnosed at a fertile age with the overall 5-year survival rate being 91.2% in women ≤44 years of age when it is found at 1A–B stage. In this scenario, testing the safety and the efficacy of fertility sparing strategies in OC patients is very important overall in terms of quality of life.

Unfortunately, the lack of randomised trials to validate conservative approaches does not guarantee the safety of fertility preservation strategies. However, evidence-based data from descriptive series suggest that in selected cases, the preservation of the uterus and at least one part of the ovary does not lead to a high risk of relapse. This conservative surgery helps to maintain organ function, giving patients of childbearing age the possibility to preserve their fertility.

We hereby analysed the main evidence from the international literature on this topic in order to highlight the selected criteria for conservative management of OC patients, including healthy BRCA mutations carriers.

## Introduction

Gynaecological cancers are relatively frequent in the female population, with a global estimated incidence of 222,700 new cases in Europe [1]. Thus, a considerable number of patients affected by gynaecological tumours are of childbearing age at diagnosis and have not completed their desire for a family. Among all ovarian cancers (OCs), 12% are diagnosed in fertile women. The overall 5-year survival rate for all OCs in women ≤44 years of age is 91.2% when it is found at stages 1A and 1B [2]. Owing to increased survival, there is a new focus on the quality of life (QoL) for cancer patients. As evidenced by a Wenzel study in which women survived lymphoma, gestational trophoblastic tumour and cervical cancer, and who were unable to procreate after cancer treatment, but who still desired fertility, experienced significant regret, so proving that the desire for reproduction is an important factor contributing to improved QoL [3]. However, compared to other tumours, the gonadotoxicity derived from medical and/or radiation treatment did not represent the main cause of infertility. In particular, for women with OC, it can be especially difficult to maintain reproductive function because the ovary is the site of primary cancer. Thus, ovaries containing ovarian follicles become a target to be treated to remove cancer cells although they should be protected for fertility.

## Psychological impact

As already demonstrated, cancer is a disease that not only causes damage and physical limitations but also causes psychological changes with negative impacts. Patients with cancer have a high rate of comorbid psychiatric disorders, as well as nonspecific psychological distress [4, 5]. Most patients with epithelial OC (EOC) experience some level of ongoing psychological distress throughout the course of their disease with particular evidence of depression and anxiety [6–8]. In a study conducted in 2006, Mc Corkle *et al* demonstrated that women with gynaecological cancer have a greater tendency towards depression, thanks to many changes in their marriages, work or financial statuses [9]. Besides, a radical reduction in QoL helps to increase an anxious and depressed state [10]. In fact, women suffering from OC experience as their first discomfort changes in their body due to radical surgery or the administration of cytotoxic therapies. Poor body image has been significantly associated with fatigue and poor sexual functioning, particularly among women who were premenopausal at diagnosis. Sexual problems may be secondary to the effects of surgery, chemotherapy or menopause, or may be due to a partner’s sexual issues or relationship problems. Women may complain of dyspareunia, loss of desire or other sexual dysfunction [11]. It’s important to identify patients with particular risk of developing sexual and psychological problems using the available questionnaire about sexual function before cancer, current sexual activity and how cancer is modifying sexual health and relationship with the partner: Brief Index of Sexual Functioning for Women or the Female Sexual Function Index [12, 13].

## Fertility-sparing methods

There are several techniques for the preservation of fertility in case of the necessity of radical surgery:

### Embryo cryopreservation

Since 1983 with the first case, embryo cryopreservation is a proven method of fertility preservation. In women with OC, this procedure can be performed only if there is no therapeutic urgency as at least 2–3 weeks is usually necessary to carry out the procedures. it is not recommended to stimulate the ovaries, after the beginning of chemotherapy, because of the proven poor ovarian response. Actually, the ovulation induction protocols from the third day of the cycle, or the one with the administration of subcutaneously injected gonadotropins for 8–14 days are effective. For this procedure, a male partner or a male gamete donor is required [14].

### Oocyte cryopreservation

Oocyte cryopreservation could be a valid option for patients with unilateral OC. Oocytes could be acquired during unilateral ovariectomy. Controlled ovarian hyperstimulation (COH) is not indicated in case of early intervention nor in prepubescent patients, and it is also not indicated in patients with granulosa cancer due to rapid hormone-dependent proliferation. The oocytes can be acquired immature or mature.

Immature oocytes are acquired without the need of stimulation and subsequently are matured *in vitro* either before freezing or after thawing [15]. In addition, immature oocytes are more resistant to cryoinjury than mature oocytes since they do not contain a metaphase spindle. Rienzi *et al* showed that to achieve successful live birth through cryopreserving oocytes, at least eight oocytes are required for patients aged <38 and more than eight oocytes in patients aged >38 [16]. Compared to embryo cryopreservation, oocyte freezing is still associated with lower pregnancy rates (4.6%–12% versus 30%–40%). In this case, no partner is needed [17].

### Ovarian cryopreservation

Ovarian cryopreservation followed by heterotopic implantation or orthotopic implantation is a technique that can be offered to patients who require early treatment and those who have hormone-sensitive tumours, in fact, no ovarian stimulation is required. It is also the only solution to offer to prepubertal patients. Ovarian tissue preservation is not an option for women with OC or at high risk of developing OC (BRCA1-2 carriers patients). However, it has been hypothesised that if the ovarian tissue is frozen when the woman is very young and at very low risk of OC, cryopreservation of ovarian tissue may still be considered [18].

However, it must be considered that surgery is not the only procedure that damages fertility in women with OC. There is also a harmful effect of chemotherapy and radiation therapy.

The most effective chemotherapeutic regimen for epithelial OC is a combination of a platinum compound and a taxane. Alkylating agents are gonadotoxic chemotherapeutic agents and have most consistently been associated with ovarian failure in a dose-dependent manner. The American Society of Clinical Oncology Clinical Practice Guideline Committee stated that women treated with high doses (≥ 5 g/m^2^) of alkylating agents have a high risk (more than 70%) to develop permanent amenorrhoea [19]. Alkylating agents determine oocytes damage via single-stranded DNA breaks and targets cells at every stage of cell cycle, preferentially on primordial follicles [20]. The impact on fertility of taxanes and platinums is an intermediate risk level (30%–70%) of amenorrhoea, whereas protocols containing antimetabolites and anthracyclines are related to a lower risk (less than 30%) [20]. Radiation is reserved for chemotherapy-resistant OCs. Lower doses of radiation therapy are used for OCs, resulting in germ cell death in the contralateral ovary in the case of unilateral oophorectomy. Germ cells are the most sensitive cells in the body to radiation and chemotherapy [21]. The reason for this high sensitivity is assumed to be related to the presence of a TAp63 molecule, a main molecule of the apoptotic pathway. TAp63, as a guardian of germ cells, decides the fate of cells depending on the intensity of DNA damage [22]. It is thought that this is a unique phenomenon in female germ cells that protect the genetic material transmission from generation to generation. c-Abl, an upstream molecule, has been shown to regulate TAp63 [23]. Through understanding the precise pathway of germ cell death and targeting the pathway, germ cells can be protected from the off-target effects of radiation and chemotherapy. Developing fertiprotective agents has long been studied to protect oocytes against radiation and chemotherapy. Gonadotropin-releasing hormone (GnRH) antagonists and agonists are used for this function. The role of temporary ovarian suppression with GnRHa during chemotherapy has been accurately studied for the past years with contrasting results [24–27]. However, recent data show the impact of this strategy in breast cancer patients [28, 29] with a significant reduction in the risk of treatment-induced premature ovarian failure and higher pregnancy rates in patients receiving GnRHa during chemotherapy [30–32].

It is clear that the choice to perform fertility sparing surgery must take into account the type of tumour and the stage.

For this reason, we reported the current available data about fertility preservation strategies in ovarian tumours, according to histotype as follows:

### Borderline tumours

Borderline ovarian tumours (BOTs) comprise 10%–20% of ovarian epithelial tumours and are typically diagnosed during reproductive years. Survival rates are about 99%, with a 70-month disease-free survival in cases of stage I tumours, and the survival rate in cases of stage III tumours is about 89% [33, 34]. While some authors recommend bilateral salpingo-oophorectomy as the initial treatment for early-stage BOT, others have also reported excellent results with more conservative treatments, including cystectomy or unilateral salpingo-oophorectomy [35]. Fertility sparing surgery was associated with a higher risk of relapse but not with increased mortality [20]. Factors associated with a higher risk of relapse after conservative surgery for early-stage BOT include age <30, bilaterality and type of surgery (cystectomy versus adnexectomy). Micropapillary histologic pattern, stage and presence of invasive implants are other well-recognised risk factors [36, 37]. Women desirous of offspring should be made to have regular intercourse after 3 months from surgery, in those women who have no partner or because they want to postpone pregnancy oocyte cryopreservation after ovarian stimulation is advised. The data in the literature suggest that it is unclear whether assisted reproductive techniques are associated with an increased risk of recurrence, but it is underlined that most recurrences (11/12) were BOT and were successfully managed by surgery [38].

Some other methods could be used in the future. Currently, we are waiting for the publication of data about two patients that underwent ovarian corticectomy as a novel technique in fertility sparing [39].

Studies concerning BOT and fertility preservation are summarised in Table 1 with the respective conception rates.

### Germ cell tumours

Malignant ovarian germ cell tumours (MOGCTs) are rare cancers (3%–5% of ovarian tumours) but they are the ones that most affect younger women—83% of cases occur in women under the age of 40 years, often in women in their teens and twenties. For this reason, the preservation of fertility is an important aspect in the management of these neoplasms [40]. Thanks to the rapid growth and early symptoms secondary to capsular distension, necrosis and haemorrhage, the tumour is frequently diagnosed in stage I unlike to epithelial OC [40]. Unilateral salpingo-oophorectomy with peritoneal staging and retroperitoneal staging if indicated, is the treatment of choice in early stage MOGCT with vertical midline incision, careful abdominal exploration with inspection and palpation of all peritoneal surfaces, multiple biopsies of peritoneum, omentectomy, with no survival difference after unilateral or bilateral salpingo-oophorectomy when MOGCT are confined to one ovary [41]. Bilateral disease is uncommon and no biopsy is advised owing to the risk of extra adhesions and impairment of ovarian reserve unless there are macroscopically suspicious areas in the contralateral ovary [42].

- Pure dysgerminoma: several authors suggest fertility-sparing treatment for all stages with a disease-free survival in 10 years of >90% and overall survival 100% [43].
- Yolk sac tumours (non-dysgerminomatous tumours): for early stage, fertility-sparing surgery is feasible, in case of a higher stage, standard-dose bleomycin, etoposide and cisplatin (BEP) chemotherapy following fertility-sparing surgery has been associated with favourable overall survival rates and no apparent compromise of fertility rates [44]. Serum alpha-feto-protein (AFP) is a reliable marker for diagnosis and may be used in clinical decision-making after surgery or for advanced disease management [45]. Even though three courses of BEP are the standard adjuvant therapy after conservative surgery for early-stage YST, some patients can also be carefully followed up without treatment if AFP after surgery declines consistently.
- Immature ovarian teratoma (non-dysgerminomatous tumours) stage 1 grade 2–3 adjuvant chemotherapy following fertility-sparing surgery has been recommended by some authors, but several studies suggest an expectant approach with BEP only in case of relapse [46]. Reproductive function, on the other hand, has been reported to be relatively good, with more than 80% of patients retaining reproductive function after chemotherapy and surgery [42]. Oocyte cryopreservation could be proposed to all adolescent patients and to all those who have not yet planned a pregnancy and a COH could also be considered after 12 months from CT.

### Malignant sex cord-stromal tumours

Malignant sex cord-stromal tumours are rare and include granulosa cell tumours (most common) and Sertoli-Leydig cell tumours; they are typically associated with a good prognosis. Most of the patients with granulosa tumours present with early-stage disease. The disease is typically indolent. Patients with stage IA or IC sex cord-stromal tumours desiring to preserve their fertility should be treated with fertility-sparing surgery. Although complete staging is recommended for all patients, lymphadenectomy may be omitted for stage IA or IC. For patients who choose fertility-sparing surgery, completion surgery should be considered after childbearing is finished. For patients with high-risk stage I tumours (tumour rupture, stage 1C, poorly differentiated tumour and tumour size >10–15 cm), observation or consideration of platinum-based chemotherapy should be indicated. Patients with surgical findings of low-risk stage I tumour (i.e. without high-risk features) should be observed. Inhibin levels can be followed if they were initially elevated [47].

Table 2 summarises studies about granulosa cell tumours and fertility preservation.

### Epithelial tumours

The standard treatment for patients in International Federation of Gynecology and Obstetrics (FIGO) stage I–II EOC is based on total hysterectomy, bilateral salpingo-oophorectomy, peritoneal sampling, omentectomy, both pelvic and para-aortic lymphadenectomy [48]. According to the available guidelines, in women wishing to maintain fertility, conservative surgery can be performed, for all grades at stage IA or IC [49, 50]. While this approach is still debated for high-risk patients (clear cell, stage> or equal IAG3) [50, 51]. In the recent ESMO and ESGO guidelines, a conservative approach is limited to G1-2 IA and IC EOC with unilateral involvement, in the case of mucinous, serous, endometrioid or mixed histotype [50, 52]. In the largest retrospective series available including 1,189 patients with early EOC, 432 of whom treated conservatively, stage IC and grade 3 were the only independent predictors of survival [53]. Fruscio *et al*, in a retrospective study, unlike Wright JD, in a 240 patients with malignant early stage/EOC treated with fertility-sparing surgery, confirmed that grade of nuclear differentiation G3 was the only predictor for survival, associated with a significant higher rate of distant recurrence (RFS: Hazard ratio [HR]: 4.2, 95% confidence interval [CI]: 1.5–11.7, *P* = 0.0067; OS: HR: 7.6, 95% CI: 2.0–29.3, *P* = 0.0032) [51]. However, patients with G3 tumours, analysed in this study, have a comparable prognosis in terms of disease-free and overall survival compared to patients with G3 neoplasia included in the ICON1/ACTION trial where all women underwent radical surgery [54, 55]. Some studies have indeed shown that in the case of macroscopically undamaged contralateral ovaries, the risk of microscopic involvement was 0%–2.5% [48, 56]. In the case of endometrial histotype, an endometrial biopsy is suggested, while in the case of mucinous histotype, appendectomy is recommended to exclude intestinal origin of tumour. Regarding the surgical procedure, the laparoscopic approach has been described as a feasible technique by several authors [48, 57]. However, tumours greater than 10 cm are probably correlated with a higher risk of rupture and spillage that may occur in 88% versus 9% through laparoscopy compared to laparotomy [58]. In a Bentivegna analysis, it has been observed that most relapses occurring after a conservative surgical approach are extra ovarian, suggesting that the preservation of one ovary is not necessarily the cause of the recurrence [59]. Moreover, the adjuvant therapy in early-stage EOC improves survival and delays recurrence in patients with IC stage, as demonstrated by a multicentre open randomised trial, with 477 patients, in fact, the group who receive adjuvant chemotherapy immediately following surgery had better overall survival (HR of 0.66, 95% CI = 0.45–0.97; *P* = 0.03) and a recurrence-free survival (HR = 0.65; 95% CI = 0.46–0.91; *P* = 0.01) [60].

Studies concerning EOCs and fertility are collected in Table 3.

Otherwise, for healthy BRCA mutated patients with elevated risk for OC, other therapeutic options are adopted.

Currently, mutation carriers should complete childbearing and then undergo a salpingo-oophorectomy around 35–40 years if *BRCA1*-mutated and 45–50 years if *BRCA2* mutated, while concurrent hysterectomy is not recommended [61–64]. Thus, for this group of patients, fertility could be impacted by two different agents: treatments of eventual cancer and prevention strategies. It has been hypothesised that carrying BRCA mutations, especially BRCA1, can be associated with decreased ovarian reserve, increased fertility-related problems and primary ovarian insufficiency that can lead to infertility and early menopause [65]. Despite the strong rationale and preclinical results suggesting this hypothesis, conflicting clinical data are available and they do not show a significant difference among BRCA carriers and non-carriers. Hence, a negative impact of carrying a BRCA mutation, mainly BRCA1 but also BRCA2 [66, 67], on women’s reproductive performance must be kept in consideration. For the lack of reproduction studies about BRCA-mutated breast cancer patients, the safety and efficacy of the different strategies for fertility preservation and the feasibility of having a pregnancy after diagnosis should be considered a research priority [68]. Approaching this group of patients, some authors suggest performing anti-Müllerian hormone measurement and antral follicle count in order to evaluate the ovarian reserve. Therefore, young adults with BRCA mutation should be counselled regarding this potential decrease of ovarian reserve [69].

In cases needing fertility preservation, oocyte cryopreservation could be an option for BRCA-mutated women who undergo surgery not at 40 years old but earlier, when the ovarian reserve and its quality are better [70]. In contrast, ovarian tissue cryopreservation is not recommended, for the increased risk of malignant transformation [71, 72]. However, two live births have been reported in the literature, with uneventful pregnancies, in BRCA-mutated breast cancer patients that underwent ovarian tissue cryopreservation. The former was 16 months after transplantation, with a previous miscarriage—the latter happened with the recovery of ovarian function 5 months after transplantation, followed by a spontaneous pregnancy 3 months later [73, 74].

Salpingectomy with delayed oophorectomy, preserving natural follicular cycle, is another option for prophylactic treatment. However, this surgery strategy is still not recommended as the primary approach [62, 63].

### Carcinosarcomas

Carcinosarcomas Malignant Mixed Müllerian Tumours (MMMTs) are rare tumours with a poor prognosis. Patients with MMMTs are not candidates for fertility-sparing surgery regardless of age.

## Future perspectives

### In vitro ovarian follicle growth

The greater risk associated with cryopreserved ovarian tissue autotransplantation is the possibility of a tumour re-implantation and dissemination, also in the contralateral ovary transplantation that could also contain tumour cells [71]. The possibility of using *in vitro* follicles could represent a chance to preserve fertility in young patients with OC; in fact, recently, mature human follicles have been successfully cultured *in vitro* to produce metaphase II stage oocytes which could be used for IVF [75, 85]. This technique does not require hormonal stimulation and can also be offered in prepubertal patients.

### In vitro ovarian follicle maturation

A future possibility to offer to young cancer patients could be follicular maturation *in vitro*. In fact, a portion of cortical ovarian cysts from patients with cancer could be treated with phosphatase and tensin homolog inhibitor or AKT activator, determining *in vitro* ovarian follicle maturation.

These follicles can be kept for future use [76].

### Protection against germ cell damage using fertoprotective agents

Fertility sparing surgical approaches in patients with early-stage OC, described above, have some limitations, including cost, time, accessibility to dedicated centres and gonadotoxicity related to procedures. Therefore, the need arises to obtain adjuvants that can inhibit or reduce the side effects of various anticancer drugs with the aim of protecting the pool of dormant follicles.

Some molecules have already been extensively studied for this role, for example, sphingosine-1-phosphate (S1P), imatinib mesylate, amifostin, tamoxifen and GnRH antagonists and agonists. There are emerging studies on the fertoprotective role of melatonin. In fact, melatonin reduces the adverse effects of chemotherapy by removing superoxide anion, hydrogen peroxide and peroxyl radical.

However, it is necessary to test agents and to develop new and efficient fetoprotective agents in the preservation of the ovarian reserve for OC patients [77].

## Actual and future perspectives for hysterectomised women (beyond uterus transplantation)

Women who must undergo hysterectomy will need to consider other options, such as surrogacy, even if they have cryopreserved oocytes. Recently, successful uterine transplantation was reported but the application of this technique in clinical practice is still limited [78]. Despite the interesting results reported by Brännström M *et al* about the use of uterus transplantation in either benign and malignant conditions, the use of high doses of immunosuppressive agents, the risk of cancer recurrence in immunocompromised patients and the possible vascular abnormalities after pelvic radiation must be considered before taking this approach in consideration to cancer patients. However, it could represent a revolutionary approach for the management of gynaecologic cancer patients with fertility preservation purposes [78].

Studies about uterus transplantation and its outcomes are summarised in Table 4.

## Conclusions

The mean 5-year survival rate for OC is 47.4% but the cancer stage at diagnosis has a strong influence on the length of survival [79]. Most women with Stage I OC have an excellent prognosis. Stage I patients with grade I tumours have a 5-year survival of over 90%, as do patients in stages IA and IB. These percentages significantly decrease for the other stages: Stage II OC has a 5-year survival rate of approximately 70%, Stage III about 39% and Stage IV only 17% [79]. Other factors impact a woman’s prognosis, including her general health, the grade of cancer and the histotype. Among women with distant disease, a higher risk of mortality was observed within the first 2 years after diagnosis for mucinous, clear cell and carcinosarcoma compared with high-grade serous, with the most striking hazard ratio observed in the first year after diagnosis for mucinous (HR 1⁄4 3.87, 95% CI 1⁄4 3.45–4.34) [80]. Cumulatively, both localised/regional and distant-stage low-grade serous and endometrioid carcinomas had the most favourable outcomes [81]. So, it is clear that different cancers need different approaches. Moreover, it is important to distinguish non-epithelial ovarian neoplasms because guidelines allow gynaecologists to perform more conservative surgery [82–84]. Therefore, since there is no scientific evidence to support a better prognosis with demolitive surgery, conservative surgery can be proposed in the initial stages with adequate counselling. Obviously, the choice must be strictly based on an accurate staging of the disease, surgically reached, and on the evaluation of prognostic factors as grading and histotype.

In the case of negative prognostic factors, chemotherapy can have a fundamental role in allowing a fertility sparing approach.

**Figure 1.**
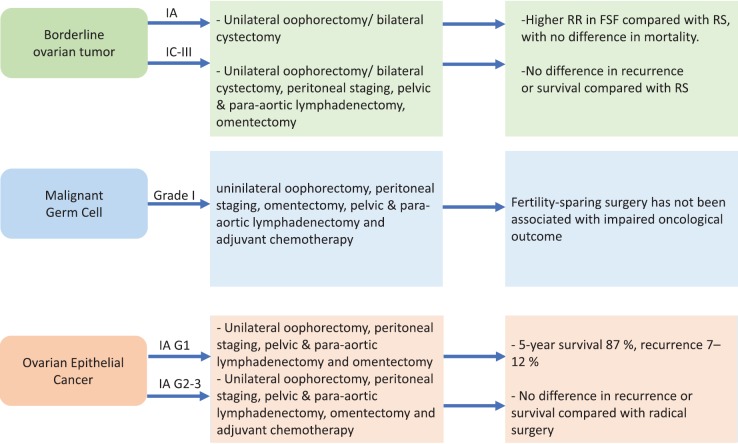
Fertility sparing strategies in OC patients. RR = recurrence rate, FSF = fertility sparing surgery, RS = radical surgery.

To sum up, it is essential to identify tumours at an initial diagnosis that allows the ovarian and uterus tissue to be maintained because only initial stages can be treated with conservative procedures reducing danger for the patients. Different approaches for each type of tumour are summarised in Figure 1.

Unfortunately, we know that there is no OC screening test but an annual pelvic ultrasound should be proposed to all women. For their predisposition to develop OC, BRCA-mutated patients are encouraged to perform closer checks and monitoring of the marker, before the oophorectomy, the most effective measure for reducing this risk.

It is true that there will be the possibility of new techniques for patients radically treated (such as uterus transplantation), but this is still very far from common clinical practice.

As long as these methods do not become habitual, early diagnosis, and thus fertility sparing surgery, is the only chance for these women to become pregnant.

## Conflicts of interest

The authors confirm that there are no conflicts of interest, financial or otherwise, associated with this article.

## Funding

The authors received no funding for the completion of this article.

## Figures and Tables

**Table 1.** BOTs and fertility.

Study	Patients	Stage	Pregnancies	Patients who conceived	Patients who attempted to conceive	Conception rate (%)
Morris *et al* 2000	43	IA–II	25	12	24	27.91
Zanetta *et al* 2001	189	IA–III	41	44	NR	23.28
Morice *et al* 2001	44	IA–III	17	14	NR	31.82
Cameatte *et al* 2002	17	II–III	8	7	9	41.18
Fauvet *et al* 2005	162	IA–III	30	21	65	12.96
Park *et al* 2009	184	IA–III	33	27	31	14.67
Uzan *et al* 2009	41	II–III	18	14	NR	34.15

NR = not reported

**Table 2.** Granulosa cell tumours and fertility.

Study	Patients	Stage	Pregnancies	Patients who conceived	Patients who attempted to conceive	Conception rate (%)
Low *et al* 2000	74	IA–IV	16	19	20	25.68
Zanetta *et al* 2001	138	IA–IC	55	28	32	20.29
Tangir *et al* 2003	64	IA–IV	47	29	38	45.31
Zanagnolo *et al* 2004	39	IA–IC	11	36	NR	92.31
Nishio *et al* 2006	30	IA–IV	4	8	12	26.67
Chan *et al* 2008	313	IA–IV	NR	29	38	9.27

NR = not reported

**Table 3.** Epithelial OCs and fertility.

Study	Patients	Stage	Pregnancies	Patients who conceived	Patients who attempted to conceive	Conception rate (%)
Zanetta *et al* 1997	56	IA–II	17	20	NR	35.71
Morice *et al* 2001	25	IA–II	3	4	4	16.00
Schilder *et al* 2002	52	IA–IC	26	17	24	32.69
Morice *et al* 2005	34	IA–IC	10	9	NR	26.47
Anchezar *et al* 2009	18	IA–IIIB	7	6	7	33.33
Schlaerth *et al* 2009	20	IA, IC	9	6	NR	30.00
Park *et al* 2008	62	IA–IIIC	22	15	19	24.19
Raspagliesi *et al* 1997	10	IA–IC	2	3	5	30.00
Borgfeldt *et al* 2007	23	IA, IC	30	15	NR	65.22

NR = not reported

**Table 4.** Uterus transplantation and outcomes.

	Pts	Age	Cause of uterus absence	Type of transplantation	Results
Fageeh W *et al* 2002	1	26	Post-partum haemorrhage	Allotransplantation from alive donor	Histerectomy for acute vascular thrombosis
Ozkan *et al* 2013	1	21	Complete müllerian agenesis	Allotransplantation from a deceased donor	Pregnancy with early miscarriage
Brännström *et al* 2014 Johannesson *et al* 2015	9	31.5	– Eight MRKH – One cervical cancer	Allotransplantation from alive donor	– Seven uteri remain viable (with mild rejection in four patients reversed with corticosteroids) – Two severe rejections caused by bilateral thrombotic arterial occlusions
Brännström *et al* 2014	1	35	MRKH	Allotransplantation from alive donor	One live birth

MRKH = Mayer Rokitanscky Küster Hauser syndrome
